# Effects of single-session cathodal transcranial direct current stimulation on tic symptoms in Tourette’s syndrome

**DOI:** 10.1007/s00221-019-05637-5

**Published:** 2019-08-28

**Authors:** Katherine Dyke, Georgina M. Jackson, Elena Nixon, Stephen R. Jackson

**Affiliations:** 1 School of Psychology, University of Nottingham, Nottingham, NG7 2RD UK; 2 Division of Psychiatry, School of Medicine, Institute of Mental Health, University of Nottingham, Nottingham, NG7 2TU UK

**Keywords:** Tourette’s syndrome (TS), Transcranial direct current stimulation (tDCS), Transcranial magnetic stimulation (TMS), Supplementary motor area (SMA)

## Abstract

Tourette syndrome is a neurodevelopmental disorder characterised by motor and phonic tics. For some, tics can be managed using medication and/or forms of behavioural therapy; however, adverse side effects and access to specialist resources can be barriers to treatment. In this sham-controlled brain stimulation study, we investigated the effects of transcranial direct current stimulation (tDCS) on the occurrence of tics and motor cortical excitability in individuals aged 16–33 years with Tourette syndrome. Changes in tics were measured using video recordings scored using the RUSH method (Goetz et al. in Mov Disord 14:502–506, 1999) and changes in cortical excitability were measured using single-pulse transcranial magnetic stimulation (spTMS) over the primary motor cortex (M1). Video recordings and spTMS measures were taken before and after 20 min of sham or active tDCS: during which cathodal current was delivered to an electrode placed above the supplementary motor area (SMA). Tic impairment scores, calculated from the video data, were significantly lower post-cathodal stimulation in comparison with post-sham stimulation; however, the interaction between time (pre/post) and stimulation (cathodal/sham) was not significant. There was no indication of a statistically significant change in M1 cortical excitability following SMA stimulation. This study presents tentative evidence that tDCS may be helpful in reducing tics for some individuals, and provides a foundation for larger scale explorations of the use of tDCS as a treatment for reducing tics.

**Electronic supplementary material:**

The online version of this article (10.1007/s00221-019-05637-5) contains supplementary material, which is available to authorized users.

## Introduction

Tourette syndrome (TS) is a childhood onset disorder, characterized by the presence of brief stereotyped behaviours of a limited duration known as tics. Tics can involve movement (motor tics) or the production of sound (phonic tics) and may become apparent in children as young as 3 years (Leckman et al. 1998). Tics can be socially alienating and physically harmful, and although tics and reactions to them vary from person to person, they have been found to influence many aspects of life including social, occupational/academic, and psychological well-being of both adults and children with TS (Conelea et al. 2011, 2013). For many individuals with TS, tics severity will decrease with age (Bloch and Leckman 2009); however, a substantial minority will continue to have tics in adulthood.

The treatment options available for individuals with TS are limited, and while behavioural interventions such as habit reversal training (HRT) are effective (Bate et al. 2011; Dutta and Cavanna 2013), they may not be readily accessible or suitable for all. As a result, one of the most common treatment options is medication, including forms of antipsychotics, which can have a number of undesirable side effects (Kurlan 2014). This makes it particularly pertinent that alternative avenues of treatment are explored, one of which may involve the use of non-invasive brain stimulation techniques such as transcranial magnetic stimulation (TMS) or transcranial direct current stimulation (tDCS). When used correctly, these techniques are safe and largely side-effect free (Bikson et al. 2016; Rossi et al. 2009).

The neurobiology of TS is yet to be fully understood; however, there is a general consensus that dysfunction in cortico–straito–thalamo–cortical networks contributes to the pathophysiology of the condition (Greene et al. 2015; Mink 2006) and that this leads to hyper-excitability within motor cortex (Orth and Rothwell 2009). One region which has often been implicated in the occurrence of tics in TS is the supplementary motor area (SMA), which has extensive connections to brain areas associated with motor control and cognitive processing (Picard and Strick 2001). The SMA has been linked to the genesis of tics in a number of multi-modal studies; for example, fMRI blood oxygen-level-dependent (BOLD) signal within the SMA has been found to increase immediately in advance of the occurrence of a tic (Bohlhalter et al. 2006) and shows a different pattern of activation during tics in comparison with voluntary movements (Hampson et al. 2009). Furthermore, increased activity within the SMA in individuals with TS has also been found using positron emission tomography (PET), and this was demonstrated to correlate with tic severity measures (Eidelberg et al. 1997). Altered levels of the inhibitory neurotransmitter GABA have also been identified within this region (measured using ultra-high-field magnetic resonance spectroscopy), and may be the basis of compensatory mechanisms through which individuals exert control over tic production (Draper et al. 2014).

Studies using TMS have also implicated the SMA as an important region for the likely generation of tics. Finis et al. (2013) found that tic-like behaviours could be evoked in healthy participants by stimulating the SMA using trains of repetitive TMS (rTMS) delivered at 5 Hz. This form of rTMS is known to lead to temporary increases in cortical excitability (Pascual-Leone et al. 1994): hence, this work suggests that elevated SMA excitability may contribute to the genesis of tics. Furthermore, the use of 1 Hz rTMS which is known to have an inhibitory effect on cortical excitability (Gerschlager et al. 2001) has been shown to successfully reduce tics in individuals with TS after 10 days of stimulation applied to the SMA (Kwon et al. 2011; Mantovani et al. 2007). These effects have been found to persist for 12 weeks after stimulation (Kwon et al. 2011), and the beneficial effects of 20 rTMS sessions have reportedly lasted as long as 6 months (Le et al. 2013). These effects are very promising, however, access to rTMS is currently limited, and attending multiple treatment sessions may be difficult for patients. tDCS offers an appealing alternative, as it is comparatively cheap, portable, and easy to administer at home. While home use tDCS is not yet common, the feasibility of this technology has recently been shown in a study of 20 participants with multiple sclerosis (Kasschau et al. 2016). Furthermore, no side effects have been reported following home use as a treatment for schizophrenia (Schwippel et al. 2017) and trigeminal nerve pain (Hagenacker et al. 2014). Hence, the exploration of tDCS as a therapeutic intervention is a viable avenue which may lead to interesting and beneficial results.

To date three small-scale studies and one pilot study have explored the use of tDCS in reducing tics. Mrakic-Sposta et al. (2008) found that tics significantly reduced following 5 days of tDCS applied to the left motor cortex in a single case study, Carvalho et al. (2015) identified a significant reduction in tics after ten sessions of cathodal tDCS were applied to the SMA, which was still present after 6 months. A pilot study conducted by Eapen et al. (2017) also targeted the SMA and reported a reduction in tics and premonitory urges in two participants following 18 sessions of cathodal stimulation. However, a recent study by Behler et al. (2018) found that only one of the three participants experienced a reduction in tic following tDCS delivered to the pre-SMA/SMA region of cortex. It should be noted that this particular study used a more intensive stimulation protocol in which participants received 2 mA cathodal tDCS for 30 min twice a day for 10 days. At face value, this may seem advantageous; however, cathodal tDCS has been shown previously to have non-linear effects and increasing the intensity has previously been found to cause an increase rather than a decrease in cortical excitability (Batsikadze et al. 2013) which may have influenced the findings.

Taken together, these findings provide support for the idea that cathodal tDCS may be useful in reducing tics; however, all were conducted using very small sample sizes (1–3 participants), and reported the effects of multiple stimulation periods. Furthermore, although two of these studies included a sham control (Eapen et al. 2017; Mrakic-Sposta et al. 2008), this was only effectively counter-balanced in one (Mrakic-Sposta et al. 2008).

In the current study, the immediate effects of a single session of cathodal/sham tDCS on tic symptoms were investigated. Change in tics was measured using short video clips of the participants taken before and after stimulation. TMS was also used as a physiological probe to explore whether changes in cortical excitability, induced by tDCS delivered to the SMA, were detectable in the primary motor cortex (M1). Two complimentary measures were used to assess this: TMS recruitment (input–output IO) curves and the response to a standard TMS stimulation (single pulse) at an intensity capable of evoking a 1 mV motor-evoked potential (MEP) response (SI 1 mV). Both these measures can be used to index excitability within a wider range of the cortex than protocols using lower intensities and are thought to reflect the strength of corticospinal projections (Chen 2000; Devanne et al. 1997). Importantly, both measured been shown previously to alter in response to tDCS stimulation delivered to M1 (Batsikadze et al. 2013; Furubayashi et al. 2008; Kidgell et al. 2013; Nitsche et al. 2005; Strube et al. 2016).

## Methods

This study gained ethical approval through the East Midlands branch of the National Research Ethics Service and was conducted in accordance with the ethical standards specified in the 1964 Declaration of Helsinki.

### Participants

A total of ten participants with a confirmed clinical diagnosis of Tourette’s syndrome (*N *= 9) or Chronic tic disorder (*N *= 1) were recruited. The mean age of participants was 22.8 years (range 16–33 years); five were male and five were female. Participants were recruited through the UK charity Tourettes action and through a local NHS clinic. Some participants had a diagnosis of additional co-occurring disorders and some were taking medication (see Table 1 for details).

**Table 1:** Participant demographics

Participant number	Sex (M/F)	Age	Tic diagnosis	Co-occurring diagnoses	Medication
1	M	23.3	TS	N/A	Clonidine
2	M	16.1	TS	Anxiety	Clonidine, Aripiprazole, Sertraline
3	M	20.5	TS	ADHD	Pentasa (*not CNS active*)
4	F	20.5	TS	N/A	N/A
5	F	18.4	TS	OCD, dyscalculia, depression	Citalopram (20 mg)
6	F	32.2	TS	N/A	N/A
7	F	33.3	TS	ADHD	Concerta, Fluoxetine
8	M	20.3	TS	N/A	Clonidine (175 mg)
9	M	20.5	TS	ADHD	Methylphenidate hydrochloride
10	F	23.1	CTD	N/A	N/A

*M M*ale, *F F*emale, *TS* Tourette’s syndrome, *CTD* chronic tic disorder, *ADHD* attention deficit disorder, *OCD* obsessive compulsive disorder

### tDCS to the supplementary motor area (SMA)

tDCS was delivered via a NeuroConn DC stimulator (GmbH, Ilmenau, Germany) with a maximum stimulation output of 4.5 mA. Stimulation was applied using surface sponge electrodes measuring 35 cm^2^. The ‘active’ electrode was placed on the area of the scalp thought to be directly above SMA in a manner that afforded bilateral stimulation. This location was identified in accordance with the previous studies (Enticott et al. 2012; Finis et al. 2013; Mantovani et al. 2007), where the EEG 10–20 system was used to identify the site located at 15% of the distance between nasion and inion, anterior to CZ. The reference electrode was placed on the right hand side of the participant’s forehead. In the cathodal condition, a 1 mA current was run between the two electrodes for 20 min, and this was ramped up for 15 s at the start of the stimulation and ramped down over 15 s at the end. These parameters have previously been found to result in changes in cortical excitability outlasting the stimulation period but up to 120 min (Batsikadze et al. 2013). In the sham condition, the current was also ramped up and down over a 15 s period, although it was only held constant at 1 mA for 30 s. This resulted in a maximum current density of .028 mA cm^2^ (1 mA/35 cm^2^) in both conditions. Participants were blind to the experimental condition; however, for practical reasons, the researcher was not. Anecdotally participants did not report feeling differences in sensation between sham and active stimulation; however, this was not systematically assessed.

### TMS measurement and EMG recording

TMS was delivered using a Magstim 200 (Magstim, Whiteland, Dyfed, UK) with a figure-of-eight magnetic coil (each winding was 70 mm in diameter). The coil was held tangentially to the scalp and oriented 45° from the midline. The optimal location for the stimulation of the contralateral FDI muscle was defined as the location over the left primary motor cortex which when stimulated consistently resulted in the largest MEP (i.e., ‘motor hot spot’). This location was used for all TMS measures.

MEPs were recorded using disposable Ag–AgCl surface electrodes attached to the right FDI muscle in a belly tendon montage. Alcohol wipes were used to prepare the skin prior to application of the electrodes. The signals were amplified and bandpass filtered (10 Hz–2 kHz, sampling rate 5 kHz) then digitised using Brainamp ExG (Brain Products GmbH, Gilching, Germany) controlled by Brain Vision Recorder (Brain Products GmbH, Gilching, Germany). Participants were encouraged to maintain their hand in a relaxed position on a table directly in front of them. Resting motor threshold (RMT) was determined, as the lowest intensity needed to yield an MEP response of 50–100 µV in the relaxed FDI muscle, in a minimum of five of ten trials.

A neuro-navigation system (Brainsight, Rogue Research Inc., Montreal Quebec, Canada) was used to track coil position in relation to the participant’s head and the location of the identified hotspot. A chin rest was used during stimulation to maintain the position of the participant’s head and minimise head movements. Participants were informed that they could take breaks if necessary and move if uncomfortable.

### IO curve measurement

IO curves were measured using TMS intensities of 100, 110, 120, 130, 140, and 150% of RMT. The order of the stimuli was randomized, controlled, and triggered via an in-house software programme (Matlab, Mathworks, MA, USA). Each intensity was tested a total of ten times and each TMS pulse was separated by an inter-stimulus interval (ISI) of 5 s (S). There was a pause every ten pulses in which the coil position was re-checked and participant comfort was assessed.

### SI 1 mV measurement

An SI 1 mV threshold was identified as the intensity needed to yield an MEP of approximately 1 mV when the coil was located over the hot spot. A total of 20 pulses were delivered to this area with an ISI of 5 s separating each individual pulse.

TMS thresholds (RMT, SI 1 mV) were not adjusted following tDCS, thereby allowing for identification of any changes in threshold through change in MEP amplitudes following stimulation.

### Video recording

Video recordings lasting 8 min were collected both before and after tDCS. During this time, the participants were instructed not to suppress their tics, and to sit and relax, and try to remain awake. The researcher waited outside the room throughout recording.

### Yale global tic severity scale

The Yale global tic severity scale [YGTSS; (Leckman et al. 1989)] was used to rate the number, frequency, intensity, complexity, and interference of motor and phonic tics that the participant had experienced during the previous week. This is a commonly used clinical assessment scale within TS research, and has been found to have good psychometric properties (Leckman et al. 1989; Storch et al. 2005). The ratings from this scale were used to generate a ‘tic profile’ for each individual which guided tic counting during analysis of the video data. The YGTSS was administered by one of the two experienced researchers; this was held constant for both sessions (sham/cathodal). For YGTSS scores and further participant details see supplementary tables 1 and 2.

### Experimental procedures

All participants completed two testing sessions which were separated by a minimum of 1 week. The order in which participants experienced stimulation (i.e., sham or cathodal stimulation) was counterbalanced. After gaining informed consent the YGTSS was administered by the primary investigator (KD) or an experienced research nurse (JF). On average, this took 15–30 min to complete.

Following completion of the YGTSS, the participant was seated directly in front of a video camera (face on) and an 8-min video recording was taken with the researcher and any other individuals (such as parents) outside of the room. The camera was set up to allow for a clear view of the participants face and upper body.

After the initial video recording was completed, the participant was seated in a comfortable chair with their head positioned on a chin rest and their right hand and forearm placed in a relaxed position on a table directly in front of them. The location of the participant’s head was then registered to a template using the Brainsight neuro-navigation system (Rogue Research Inc., Montreal Quebec, Canada) and disposable electrodes were attached to the hand. Following this, the hotspot for FDI stimulation was identified and mapped onto the template brain to aid coil localization. RMT was then defined before the measurement of IO curves. Following this SI 1 mV threshold was measured and data was collected from 20 pulses delivered at this intensity.

After the first session of TMS, the approximate location of the SMA was measured using the method previously described. This was marked in pen to aid placement of the tDCS electrode. The saline soaked sponge covered tDCS electrodes were then placed over this mark and over the right side of the forehead. These were attached using a rubber band and elasticated bandage. Participants remained seated during 20 min of sham or active stimulation, following which the electrodes were removed and re-registration of the participants head was performed using the neural navigation software. The hotspot was then checked using the previously sampled location as a starting reference. IO curves and SI 1 mV measures were then taken using the same intensities as in the pre-tDCS condition. Throughout the TMS and tDCS protocols, participants were able to watch wildlife documentaries. This was done in an attempt to maintain similar levels of arousal and attention during stimulation.

**Fig. 1:**
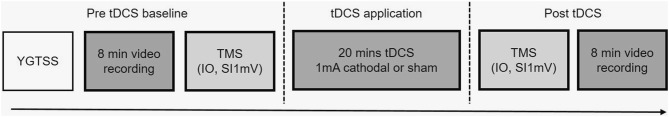
Schematic of experimental procedure

After the second session of TMS, another 8-min video recording was made with the participant alone in the room. Following this, the participants were thanked and received financial compensation for their time and inconvenience. Approximately 15–25 min elapsed between tDCS ending and the second video being recorded. The whole procedure was completed twice for each participant in a counter-balanced fashion, with at least 1 week separating each testing session, see Fig. 1 for summary of experimental procedure.

### Tic coding procedure

Prior to tic coding, the videos were anonymised. Therefore, coders scored all videos while blind to the experimental condition. A list of potential tics was generated (i.e., a ‘tic profile’) to aid tic identification for each participant using the tic-type subscale of their response to the YGTSS. Videos were played using VLC media player, the advanced tools options were used to allow videos to be slowed down and played frame by frame. Where possible a continuous 5-min segment was sampled from the 8-min videos. This sample was taken from the 2-min point onwards to allow participants to relax and become familiar with the situation.

Each video recording was scored using the Modified Rush Video Scale (Goetz et al. 1999). The scale has five components which are as follows: number of body areas, motor/phonic tic frequency [scored as tics per minute (TPM)] and motor/phonic tic severity, each of which have been found to correlate well with comparable items on the YGTSS (Goetz et al. 1999). The total impairment score, calculated from the Rush by summing the five measured components, has also been found to correlate with the ‘impairment subscale’ of the YGTSS (Goetz et al. 1999) which measures the overall impact of having tics on quality of life. Each component on the Rush is typically scored on a scale of 0–4; however, for the purposes of this study; it was only possible to score 0–3 on the body areas component. This is because tics were only counted from the upper body and face, meaning the maximal amount of body areas was 5, which corresponds with a rating of 3 on the scale. As a result, the maximal score possible on the Rush in this study was 29 rather than 30. The scores from each minute segment were combined to calculate the mean Rush score for each video clip.

### Assessment of inter-rater reliability

The 5-min video segments were first analysed by the primary investigator (KD) who then trained two secondary coders (ER and KF). Training was conducted using 1-min video segments taken from the start of recordings (these were not included in the later analysis). Once the coders were familiar with the distinct tics of each participant, they were given 2 min segments to rate using the Rush. Each coder (KF and ER) scored half of the participants, resulting in 40% of the total data being reviewed twice.

Inter-rater agreement between the primary investigator and secondary coders was assessed for each of the double scored 2 min video clips. For each minute segment, the lower score was divided by the higher score to calculate the difference. The average agreement across the 2 min was then calculated from this value. This revealed 85% agreement on the Rush total impairment score between coders KD and KF (range 71–96%), and 86% agreement between coders KD and EF (range 67–100%).

### Input–Output curves and SI 1 mV

Peak-to-peak MEP amplitudes were estimated using in-house Matlab programmes (Mathworks, MA, USA). All trials in the 500 ms period prior to MEP were visually inspected and any trials in which there was evidence of pre-contraction of the FDI muscle were excluded from analyses. Mean percentage excluded per condition (for pre/post, sham/cathodal and IO curve/SI 1 mV) ranged from 2.2 ± 3.25 to 7.5 ± 12.75.

IO curve measurements were estimated by calculating the median intra-individual MEP amplitudes for each TMS intensity value (i.e., 100–150% of RMT). Median values were calculated rather than the mean to limit the effect of outliers within individual data. Linear fits were then applied to the resultant values (mean *R*^2^ = .87). Four-parameter sigmoidal fits were also applied to the IO curve data. Grubbs outlier tests with an alpha level of .001 identified no outliers for linear slope fits. For sigmoidal fitting two data sets for maximal slope were identified as outliers and one data set for slope plateau. Statistical analysis of sigmoidal slopes revealed similar findings to those of linear fits, and these are not detailed here but are available upon request. In addition to slope fits, we also calculated the area under the curve using the Matlab (Mathworks, MA, USA) function ‘trapz’.

Rush, IO curve and SI 1 mV data showed no clear skew and approximately normal distribution of data points as seen in QQ plots and Shapiro–Wilik tests. Therefore, parametric statistics (repeated measures ANOVAs and paired samples *t* tests) were deemed appropriate for the analysis. To further strengthen interpretation of the data we also report Baysian statistics. Bayes factors (BF10) were calculated using JASP (JASP-team, 2016). Bayes Factors above 1 suggest support for the alternative, while below 1 show support for the null. Measures of areas under the IO curve slope were not normally distributed and were assessed using Wilcoxon signed-rank tests.

## Results

### Video monitoring of tics: Rush

**Fig. 2:**
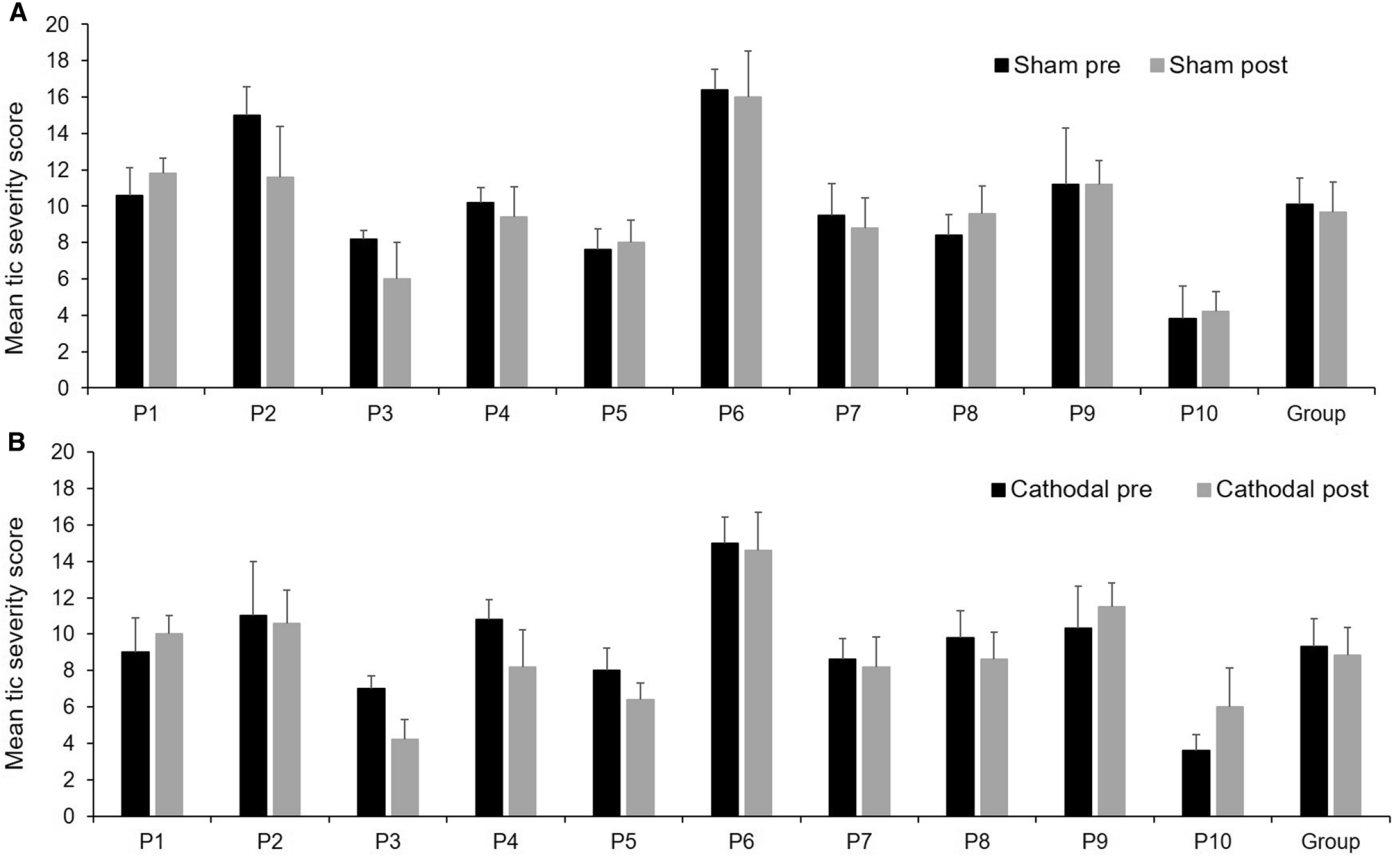
Mean ± SD tic severity score using Rush scale before and after sham stimulation (**a**) or cathodal stimulation (**b**)

A repeated measures ANOVA calculated using the *total impairment score* (body areas + tic frequency + tic severity) revealed a significant main effect for tDCS type (cathodal/sham) *f*(1,9) = 6.70 *p* = .03, *η*^2^ = .43. The main effect of time (pre/post) was not significant *f*(1,9) = 1.25, *p *= .29, *η*^2^ = .12; and there was no significant interaction between these two factors *f*(1,9) = .009, *p* = .93, *η*^2^ = .001. Paired samples *t* tests (two-tailed) revealed no significant baseline differences between the sham and cathodal conditions *t*(9) = 1.65, *p *= .13, *d* = .52. However, there was a significant difference between the post-sham (*M *= 9.66, *SD *= 3.29) and post-cathodal (*M *= 8.83, *SD *= 3.00) conditions *t*(9) = 2.35, *p *= .04, *d* = .74, indicating a significantly lower tic impairment score post-cathodal stimulation. Average change in total impairment scores can be seen for each individual in Fig. 2.

Bayesian repeated measures ANOVAs revealed evidence in favour of a difference between conditions (*BF*_10_ = 2.45) but not time (*BF*_10_ = .06). The interaction between condition and time favoured the experimental hypothesis (*BF*_10_ = 1.61).

### Transcranial magnetic stimulation: IO curve

**Fig. 3:**
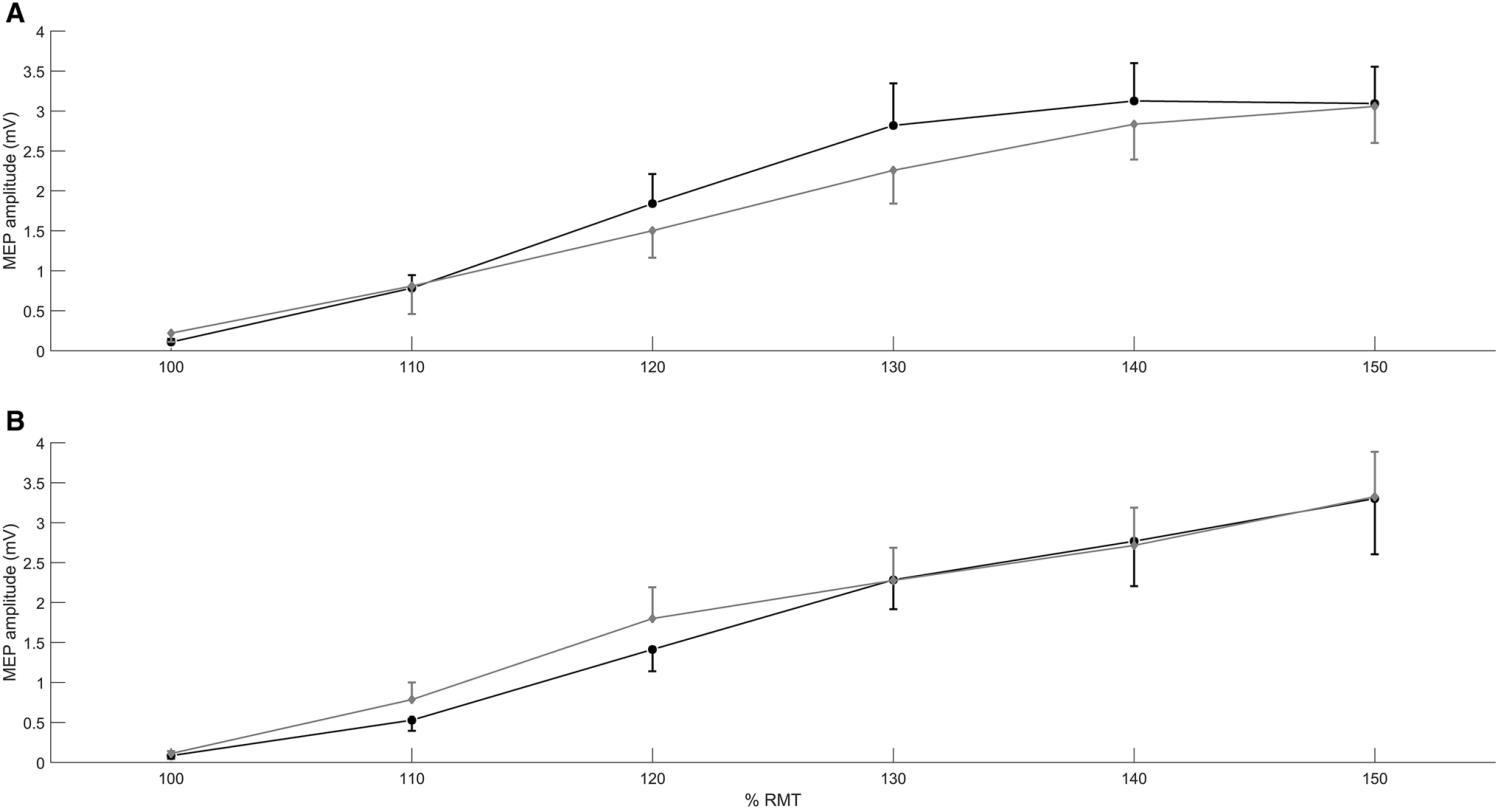
Mean ± SEM IO curve slots. **a** before (black) and after (grey) sham tDCS, **b** before (black) and after(grey) cathodal tDCS

Paired sample *t* tests revealed no significant differences between IO curve slopes measured in the pre-sham (*M *= 65.53, *SD *= 30.71) and pre-cathodal (*M *= 67.65, *SD *= 46.14) conditions *t*(9) = − .17, *p *= .87, *d* = .05. A repeated measures ANOVA was calculated in which time (pre/post) and tDCS type (sham/cathodal) served as independent factors. IO curve slope was entered as the dependent variable. The analysis revealed no significant main effects of tDCS type *F*(1,9) = .11, *p *= .75, *η*^2^ = .01 or time *F*(1,9) = 1.16, *p *= .31, *η*^2^ = .11 and no significant interaction between these two factors *F*(1,9) = .030, *p *= .87, *η*^2^ = .003. Data showing average IO curve plots for each condition can be seen in Fig. 3.

Bayesian repeated measures ANOVAs supported the null hypothesis for condition (*BF*_10_ = .03), time (*BF*_10_ = .37) and interaction between the two (*BF*_10_ = .01).

Wilcoxon signed-rank tests revealed no statistically significant differences between baseline conditions for area under the curve (*p *= .44). There was also no statistically significant difference between pre- and post-sham (*p *= .33) or pre- and post-cathodal (*p *= .65) conditions.

### Transcranial magnetic stimulation: SI 1 mV data

Paired sample *t* tests revealed no significant difference between baseline MEP amplitude evoked in the SI 1 mV condition in the cathodal (*M *= 1302.1*, SD *= 278.4) and sham conditions (*M *= 1467.53, *SD *= 514.7), *t*(9) = − .89, *p *= .40, d = − .28. Repeated measures ANOVA revealed no significant effects of tDCS type *F*(1,9) = .67, *p *= .43, *η*^2^ = .07; no significant effect of time *F*(1,9) = 2.60, *p *= .14, *η*^2^ = .22 and no significant interaction between the two factors *F*(1,9) = .101, *p *= .75, *η*^2^ = .01.

Bayesian repeated measures ANOVAs favoured the null hypothesis over the experimental for condition (*BF*_10_ = .41) time (*BF*_10_ = .90) and the interaction (*BF*_10_ = .37).

### Associations between TMS data and Rush scores

A series of multiple stepwise regression analyses were conducted to explore potential relationships between TMS measures of cortical excitability and total tic impairment scores following tDCS. Baseline IO curve slope, post-IO curve slope, baseline SI 1 mV and post-SI 1 mV were entered into the model as predictors of tic impairment score following cathodal stimulation. The analysis suggested that a large proportion of the variance in post-cathodal impairment score can be explained by the slope of the IO curve measured at baseline (*t* = 4.46, *p* = .003) and post-stimulation (*t* = − 3.37, *p* = .01); *R*^2^ = .74, *F* = 10.18, *p* = .009. No other variables contributed significant variance. When the same analysis was run for the sham condition, no significant predictors were found (*R*^2^ = .29, *F* = 1.4, *p* = .31).

## Discussion

This study investigated the effects of applying 1 mA cathodal or sham stimulation to the SMA for 20 min in individuals with Tourette’s syndrome. The impact on tic expression and cortical excitability were explored.

### Effects of single sessions of tDCS on tics

The effects on tics were assessed using short video clips taken before and after tDCS. The data was analysed using the Rush method (Goetz et al. 1999) in which tic frequency, tic severity and the amount of body areas involved are considered. The individual components of the Rush scale were not found to significantly differ following stimulation; however, the total impairment score (tic frequency + severity + number of body areas) revealed an effect. Specifically, although there were no statistically significant differences between sham and cathodal conditions at baseline, total impairment scores were significantly lower in the cathodal condition following stimulation. Although potentially interesting, in the absence of a time and stimulation interaction effect, this finding should be treated with caution.

Unlike previous work (Carvalho et al. 2015; Mrakic-Sposta et al. 2008), this study finds limited evidence to suggest that cathodal tDCS has a substantial impact on tics in individuals with TS. It is likely that this reflects methodological differences regarding the amount of cathodal tDCS sessions participants experienced. In their single case study, Carvalho et al. (2015) reported a significant 21% reduction in self-reported measures of tics (YGTSS) following five sessions of stimulation; this increased to 41% following ten sessions. Mrakic-Sposta et al. (2008) also reported an increase in effect as time went on, with the effects of 5 days of cathodal stimulation being significantly stronger than those occurring on the 4 previous days or following sham stimulation. This was found in both self-report measures (YGTSS and visual analogue scale) and from tic counts using the Rush protocol (Goetz et al. 1999). Yet, despite this, it was somewhat surprising to see no change following a single session, as single sessions of tDCS have repeatedly been shown to modulate cortical excitability and task performance in healthy adults. In particular, the effects of 20 min 1 mA stimulation have been found to produce changes in cortical excitability lasting up to 120 min after stimulation cessation (Batsikadze et al. 2013), far longer than the time elapsed between tDCS and subsequent TMS/video measures in this study. It may be that the nature of tics in TS requires more prolonged interventions and that these methods can influence tics in ways that a single session cannot. Exactly why this could be the case is unclear. The mechanisms underlying the effects of tDCS are still not fully understood, and the underlying effects of longer courses of stimulation and at areas outside of M1 remain even more enigmatic. However, it has been speculated that longer term effects may depend on modulations of the strength of underlying synaptic connections (Stagg and Nitsche 2011).

Although the amount of stimulation given is an important consideration, it should also be acknowledged that individual differences in response to stimulation may also have contributed to the effects seen here and the disparity with the previous work. tDCS-induced changes in cortical excitability have been found to vary from individual to individual (Dyke et al. 2016; Horvath et al. 2016; Lopez-Alonso et al. 2015; Strube et al. 2016; Wiethoff et al. 2014), and therefore, it seems highly likely that tDCS-induced changes in tics will also be variable. For the therapeutic potential of tDCS and other forms of non-invasive brain stimulation to be maximised, individual variation in response to stimulation must be further explored and whenever possible, treatment should be tailored towards this. Understandably, the majority of intervention studies with TS and wider conditions focus on change in clinical measures; however, it is critically important that physiological changes are also studied. While this may be particularly challenging outside of the primary motor cortex, the results of the regression analysis within this study suggest that there is still important information to be gained from these measures. The association found between IO curve measures and total impairment scores hint at a potentially important relationship between levels of cortical excitability and tics following cathodal stimulation applied to the SMA. If replicated, this type of information could aid identification of those who could benefit from this particular for of non-invasive brain stimulation.

While this study represents the largest of its kind to date, it is clear that larger scale studies will be required to further explore issues such as individual variability within therapeutic contexts. This is likely to be particularly important in heterogeneous groups. The participant sample used within this study all had a core diagnosis of a tic disorder (Tourette syndrome or chronic tic disorder); however, they vary in terms of additional diagnosis and medication. While this is experimentally challenging, it is the reality of many clinical groups and hence it is critical that such individuals are included in studies. We strongly feel that for non-invasive brain stimulation methods such as tDCS to be developed into clinically relevant treatments that the emphasis must be on exploring effects at an individual level. Unfortunately, our sample is not sufficiently large for this type of exploration, which is why we stress the importance of future studies using large, inclusive samples of the population of interest to explore this issue. Multi-modal approaches including neuroimaging techniques are also likely to prove insightful into understanding those who may benefit the most from these methods.

### Effects of cathodal tDCS applied to the SMA on cortical excitability at M1

Although the main focus of this study was exploration of the effects of cathodal tDCS on the occurrence of tics in TS; changes in MEPs measured from M1 were also assessed. To our knowledge no studies have investigated changes in MEP amplitude after tDCS stimulation of the SMA; however, studies using facilitatory rTMS protocols (5 Hz and 10 Hz) have repeatedly shown that modulation of excitability at the SMA can influence MEP amplitudes (Laviolette et al. 2013; Matsunaga et al. 2005; Raux et al. 2010). In the current study, tDCS was shown not to have any significant effect on cortical excitability at M1, as measured by IO curve and SI 1 mV measures. However, there are a number of reasons why the effects of tDCS reported here may differ from previous reports using rTMS, many of which may also explain the lack of tDCS-induced reductions in tic frequency. First, it is important to note that this study was conducted in participants with TS, whom by definition are likely to have functional/structural neuroanatomical differences (Draper et al. 2014; Jackson et al. 2011; Worbe et al. 2010, 2012) to the healthy populations studied by Laviolette et al. (2013), Matsunaga et al. (2005) and Raux et al. (2010). Research investigating immediate responses to plasticity inducing protocols in TS is sparse; however, there is some evidence that it may be altered. For example Brandt et al. (2014) found LTP type effects were not induced by paired associative stimulation (PAS) in TS when delivered using standard stimulation parameters although the predicted effects were observed in a matched control group of typically developing individuals.

Second, the focality and depth of current penetration are different between the two techniques, with traditional tDCS techniques being particularly inferior regarding focality (Priori et al. 2009). It is, therefore, possible that tDCS did not influence the SMA to the same degree as rTMS protocols.

Third, while it is possible to individualise rTMS protocols based upon each individual’s resting motor threshold, this is not currently possible for tDCS, hence the intensities used may not have been optimal across all individuals. Finally, the effects of cathodal tDCS are not reliable, and multiple studies have failed to obtain significant reductions in motor excitability (as measured by decreases in MEP amplitude) following cathodal tDCS delivered to M1 (Dyke et al. 2016; Strube et al. 2016; Wiethoff et al. 2014).

## Conclusions

In summary, the effects of 1 mA cathodal stimulation of the SMA were compared with sham stimulation in individuals with Tourette syndrome. Changes in tics were measured using video recordings and carefully quantified using the Rush protocol (Goetz et al. 1999); cortical excitability was assessed using IO curves and SI 1 mV. A small but significant difference between post-sham and post-cathodal conditions was found, with the predicted larger reduction in tics being observed after cathodal tDCS. Although this is a promising finding, and in line with previous work (Carvalho et al. 2015; Mrakic-Sposta et al. 2008), this result should be treated with some caution and warrants further investigation with larger sample sizes which would allow for further exploration of factors which predict response to stimulation cathodal tDCS did not significantly influence cortical excitability as measured by the magnitude of MEPs recorded from TMS delivered to the hand area of M1. There are a number of reasons why this might have occurred including inadequate parameter selection/electrode placement and individual variability in response to stimulation. Overall, the study does not provide strong evidence at a group level for the immediate effects of cathodal tDCS applied to the SMA; however, neither can it be seen as a strong case against the use of tDCS in Tourette syndrome. We suggest that there is a need for individualized protocols, increased sample sizes, and longer term studies to fully explore the therapeutic potential of tDCS in reducing tics.

## Electronic supplementary material

Below is the link to the electronic supplementary material.

Supplementary material 1 (DOCX 13 kb)

Supplementary material 2 (DOCX 14 kb)
