# Supplementary material for: Effects of single-session cathodal transcranial direct current stimulation on tic symptoms in Tourette’s syndrome
Source: Exp Brain Res. 2019 Aug 28;237(11):2853–63. doi: 10.1007/s00221-019-05637-5 (PMC6794240; doi:10.1007/s00221-019-05637-5)
Supplement: Supplementary file 1 — Supplementary material 1 (DOCX 13 kb) [file 221_2019_5637_MOESM1_ESM.docx]

| **Cathodal** | | | Tic Severity | | | | Pattern of current tics | |
| --- | --- | --- | --- | --- | --- | --- | --- | --- |
| Participant  number | Sex  (M/F) | Age  (y/m) | Global | Motor | Phonic | Clear presence of complex tics (Y/N) | | Characteristic motor/ phonic tics (upper body only) |
| 1 | M | 23.3 | 31 | 8 | 19 | Yes | | *Motor:* eye blink, eyebrows raise, mouth movement, head nodding/jerk, shoulder shrug, facial grimace. *Phonic:* throat clearing, coprolalia. |
| 2 | M | 16.1 | 60 | 16 | 14 | Yes | | *Motor:* eye blink, mouth/jaw movement, head jerk/movement, abdominal tensing, shoulder shrugging, facial grimace. *Phonic:* throat clearing, grunting, mm & ch sounds, occasional words. |
| 3 | M | 20.5 | 55 | 22 | 13 | Yes | | *Motor:* eye blink, squeeze eyes shut, eye movement, eyebrow raise, mouth movement, head jerks/ movement, shoulder shrug. *Phonic:* throat clear, sniff, sounds with lips. |
| 4 | F | 20.5 | 35 | 17 | 0 | Yes | | *Motor:* eye movement, head jerk/movement, shoulder movement (sometimes related to arm & hand gestures). *Phonic:* none. |
| 5 | F | 18.4 | 39 | 15 | 14 | Yes | | *Motor:* eye blink, eye movement, eyes squeezed shut, nose movement, head jerk/movement, facial grimace. *Phonic:* sniffing, whistling, chirp sounds, syllables (ch, th), spitting sound. |
| 6 | F | 32.2 | 9 | 7 | 0 | Yes | | *Motor:* nose movement, mouth movement, abdominal tensing, facial grimace. *Phonic:* none. |
| 7 | F | 33.3 | 47 | 18 | 16 | Yes | | *Motor*: eye blink, eye movement, eyebrows raise, nose movements, mouth movement, facial grimace. *Phonic:* throat clearing, coughing, clicking with tongue, echolalia. |
| 8 | M | 20.3 | 60 | 16 | 9 | No | | *Motor:* eye blink, eye movement, nose movement, abdominal tensing.  *Phonic:* throat clearing, grunting. |
| 9 | M | 20.5 | 35 | 18 | 12 | No | | *Motor:* eye blink, eyebrow raise, eye movements, head jerks/ movement, shoulder movement. *Phonic:* throat clearing, sniffing. |
| 10 | F | 23.1 | 55 | 15 | 0 | Yes | | Motor: eye blink, mouth movements, head jerk/movement, shoulder shrugging, trunk movement. *Phonic:* none. |
| Average *Global*: 41.9 ± 17.2; Average *Motor*: 15.0 ± 4.4; Average *Vocal*: 9.7± 7.17 | | | | | | | | |
