# Supplementary material for: Effects of single-session cathodal transcranial direct current stimulation on tic symptoms in Tourette’s syndrome
Source: Exp Brain Res. 2019 Aug 28;237(11):2853–63. doi: 10.1007/s00221-019-05637-5 (PMC6794240; doi:10.1007/s00221-019-05637-5)
Supplement: Supplementary file 2 — Supplementary material 2 (DOCX 14 kb) [file 221_2019_5637_MOESM2_ESM.docx]

| **Sham** | | | | Tic Severity | | | | Pattern of current tics | |
| --- | --- | --- | --- | --- | --- | --- | --- | --- | --- |
| Participant  number | Sex  (M/F) | Age  (y/m) | Global | | Motor | Phonic | Clear presence of complex tics (Y/N) | | Characteristic motor/ phonic tics (upper body only) |
| 1 | M | 23.3 | 29 | | 9 | 17 | Yes | | *Motor*: eyebrow raising, mouth movement, head nodding/jerks, shoulder shrug, facial grimace. *Phonic*: grunting, throat clearing. |
| 2 | M | 16.1 | 66 | | 17 | 9 | Yes | | *Motor:* eye blink, eyebrow raising, mouth/jaw movement, head jerks/movement, shoulder shrugging, facial grimace. *Phonic:* throat clearing, grunting, ‘hm’ & ‘mm’ sounds, occasional words. |
| 3 | M | 20.5 | 48 | | 18 | 10 | Yes | | *Motor*: eye blink, eyebrow raising, mouth/jaw movement, head jerk, head nodding, shoulder shrug, trunk movement, facial grimace. *Phonic*: throat clearing, sniffing, sounds with lips. |
| 4 | F | 20.5 | 27 | | 18 | 0 | Yes | | *Motor:* eye movement, head jerk/movement, shoulder movement (sometimes related to arm/hand movements). *Phonic*: none |
| 5 | F | 18.4 | 24 | | 11 | 14 | Yes | | *Motor*: eye blink, eye movement, eyes squeezed shut, nose movement, head jerk/movement, facial grimace. *Phonic*: coughing, throat clearing, whistling, ‘ch’, ‘ff’, ‘th’ and ‘sh’ sounds, chirping sounds, blowing. |
| 6 | F | 32.2 | 7 | | 6 | 0 | Yes | | *Motor: eye blink,* nose movement, mouth movement, facial grimace.  *Phonic:* none. |
| 7 | F | 33.3 | 52 | | 19 | 18 | Yes | | *Motor*: eye blink, eye movement, eyebrow raise nose movement, mouth movement, facial grimace. *Phonic*: throat clearing, coughing, clicking with tongue, echolalia, paralalia, a few words. |
| 8 | M | 20.3 | 60 | | 16 | 9 | No | | *Motor:* eye blink, eye movement, nose movement, mouth movement, trunk.  *Phonic:* throat clearing, sniffing, grunting. |
| 9 | M | 20.5 | 39 | | 17 | 12 | No | | *Motor:* eye blink, head jerk/ movement, shoulder movement. *Phonic:* throat clearing, sniffing. |
| 10 | F | 23.1 | 59 | | 16 | 0 | Yes | | *Motor:* eye blink, head jerk/movement, mouth, shoulder shrug, trunk movement. *Phonic:* none. |
| Average *Global*: 41.1 ± 19.06; Average *Motor*: 14.7 ± 4.42; Average *Vocal*: 8.9 ± 6.85 | | | | | | | | | |
